# Mid-to-Late-Life Anxiety and Sleep during Initial Phase of COVID-19: Age- and Sex-Specific Insights to Inform Future Pandemic Healthcare

**DOI:** 10.3390/brainsci14040346

**Published:** 2024-03-30

**Authors:** Ashley F. Curtis, Sadhika Jagannathan, Madison Musich, Mary Beth Miller, Christina S. McCrae

**Affiliations:** 1 College of Nursing, University of South Florida, Tampa, FL 33612, USA; 2 Department of Behavioral Medicine and Psychiatry, West Virginia University, Morgantown, WV 26506, USA; sadhika.jagannathan@hsc.wvu.edu; 3 Department of Psychological Sciences, University of Missouri-Columbia, Columbia, MO 65211, USA; mmusich@mail.missouri.edu; 4 Department of Psychiatry, University of Missouri-Columbia, Columbia, MO 65212, USA; millmary@health.missouri.edu

**Keywords:** COVID-19, anxiety, sleep, sleep efficiency, total sleep time, sleep quality, sex and gender, aging, older adults, pandemic, mental health

## Abstract

This study examined associations between COVID-19-related anxiety and sleep in middle-aged and older adults and tested whether these varied by age or sex. In June/July 2020, middle-aged/older adults aged 50+ (*n* = 277, 45% women, M_age_ = 64.68 ± 7.83) in the United States completed measures of sleep and COVID-19-related anxiety. Multiple regressions examined whether anxiety was independently associated with or interacted with age or sex in its associations with sleep health, controlling for age, education, medical conditions, sleep/pain medication use, and COVID-19 status. Greater COVID-19 anxiety was associated with worse sleep quality and daytime dysfunction. COVID-19-related anxiety interacted with age (not sex) in associations with total sleep time and sleep efficiency. Greater anxiety was associated with shorter total sleep time and lower sleep efficiency in oldest-older adults (~73 years old) and youngest-older adults (~65 years old) but not middle-aged adults (~57 years old). In mid to late life, older adults may be most vulnerable to the impact of COVID-19-related anxiety on sleep health. Social and behavioral (e.g., knowledge on age-related vulnerability to COVID-19 risk/morbidity/mortality, uncertainty, and changes to daily routines) and physiological factors (sleep disruption and age-related autonomic dysfunction) may underlie these associations. Interventions that mitigate negative pandemic-related psychological and sleep outcomes may be particularly relevant for older adults.

## 1. Introduction

Similar to other pandemics (e.g., H1N1 [1]), COVID-19 led to a global mental health burden [2]. Studying the factors associated with this burden will increase understanding regarding who may be most vulnerable to the psychological and behavioral impact of not only COVID-19 but future global health crises. Early in the pandemic, many individuals developed anxiety surrounding infection [3], in part due to the medical complications associated with the disease and the potential for fatality (7 million deaths reported as of May 2023; [4]). Given the known links between generalized anxiety and sleep health [5], it is critical to assess the link between anxiety specifically related to COVID-19 and aspects of sleep health. These evaluations are especially important for aging adults in mid to late life, who, in comparison to younger adults, already experience worse sleep health (e.g., greater prevalence of insomnia symptoms such as falling and/or staying asleep and clinical insomnia disorder in which symptoms are experienced for 3+ months and coupled with daytime dysfunction [6,7]), as well as more medical comorbidities that exacerbate the risk of health-related consequences of COVID-19 [8]. Further, there have been sex differences reported regarding the morbidity and mortality of COVID-19 [9,10], generalized anxiety experienced during the pandemic [11,12], and behavioral factors specifically associated with COVID-19 [13,14]. Therefore, research exploring whether sex differences exist in the COVID-19 anxiety and sleep relationship is also warranted. Taken together, increased understanding of the age- and sex-specific impact of anxiety tied to COVID-19 and sleep health will inform who may be most vulnerable to disrupted mental and behavioral health in the ongoing and future pandemics, as well as point to sex- and/or age-specific treatment targets to mitigate these negative consequences.

In the early stages of the pandemic, almost half of Americans were anxious about acquiring COVID-19 [15]. Age differences in these anxiety levels have also been reported. For instance, studies showed that, in the early stages of lockdown (in February and March 2020), relative to older adults, younger Chinese adults (less than 35 years of age) and younger to middle-aged Spanish adults (aged 18–59 years of age [16]) had greater generalized anxiety levels. Similarly, a study [17] conducted in April–May 2020 in adults across the lifespan (aged 18–75+ years) living in Latin and Caribbean countries showed that younger adults aged 18–24 showed the highest prevalence of generalized anxiety symptoms compared to all other ages. Sex differences in COVID-19 mental health outcomes also exist, with several studies conducted in early 2020 showing that younger [11] and middle-aged women [18,19,20] were more likely than men to report increased general stress/anxiety since the beginning of the pandemic. Other studies in the early stages of the pandemic showed that, across the lifespan, there was a general pattern of worse generalized anxiety [17] and post-traumatic stress [21] in women relative to men.

Sleep disruptions related to COVID-19 have also been reported, but the findings regarding age and sex differences are inconsistent. In the very early stages of the pandemic (February 2020), 18% of Chinese adults (aged 6–80 years) reported poor sleep quality (indicated by a Pittsburgh Sleep Quality Index (PSQI) score of 7 or higher), and the levels were not impacted by age or sex. A large-scale meta-analysis of international data from November 2019 to July 2020 reported higher cumulative prevalence of sleep problems of 36% (as measured by the PSQI, Insomnia Severity Index (ISI), and other validated questionnaires) and found that men and older adults were most vulnerable to sleep dysfunction. Another study [11] in the United States found that, in adults across the lifespan, those with reductions in total sleep time during COVID-19 showed higher overall insomnia symptoms (as measured by the ISI) and overall sleep quality (as measured by the PSQI). However, sex differences in these patterns were not observed. Another study [22] of adults (aged 17 years and older) in the United Kingdom showed that women reported more reductions in sleep during early COVID-19. Interestingly, a study [23] in Italian adults showed overall worse sleep quality (assessed via PSQI) and insomnia severity (assessed via ISI) in women than men in March 2020. However, only men showed worsening of both sleep metrics one month later, whereas women remained stable for global sleep quality but showed a reduction in insomnia severity [23].

There is a growing area of research examining associations between mental health outcomes (including anxiety) and sleep health during COVID-19, but there are limited studies examining age and sex differences in these relationships. One study [11] in the United States showed that, in adults across the lifespan, state anxiety was higher in those who reported greater insomnia symptoms (as assessed by the ISI) relative to those who reported decreased or unchanged insomnia symptoms, but sex and age differences in these associations were not explored. Another study [24] found that, in adults (aged 18–82 years old) living in the United States in July/August 2020, the overall negative impact and/or experience of COVID-19 (measured using the Complementary And Integrative Research Pandemic Impact questionnaire and included an assessment of COVID-19 exposure, stress, and related impact of COVID-19 and personal growth due to COVID-19) was associated with worse overall insomnia symptom severity (as measured by the ISI). Age and sex did not moderate this relationship, but whether these factors potentially moderate the impact of COVID-19-specific anxiety and sleep was not tested. Additionally, evaluations in Chinese adults across the lifespan (18–52 years of age) found that greater post-traumatic stress symptoms were associated with worse sleep quality and shorter duration of sleep, but no sex differences or age differences in the associations were reported [21]. Taken together, inconsistent and limited age and sex investigations, as well as a lack of investigations in middle-age and older adults, prompt the need for more examinations on how COVID-19-specific anxiety impacts sleep health in aging adults.

The goal of this study was to determine whether anxiety specifically related to COVID-19 is associated with aspects of sleep health (including global sleep quality, subjective sleep quality ratings, total sleep time, sleep efficiency, and daytime dysfunction) in middle-aged and older adults during the plateau phase (July–August 2020) of the COVID-19 pandemic. Additionally, we also tested whether these associations were moderated by age or sex. First, we hypothesized that higher levels of COVID-19 anxiety would be associated with worse sleep health. Second, we hypothesized that the associations would be strongest in older adults relative to middle-aged adults. Third, given previous work showing sex differences in both levels of COVID-19 anxiety [17,21] and sleep quality [22,23], we did hypothesize that sex would moderate the COVID-19-related anxiety and sleep relationship. However, given the limited research on sex differences in COVID-19 mental health outcomes and sleep associations, we did not pose specific hypotheses regarding whether the associations would be stronger in men or women.

## 2. Methods

### 2.1. Participants

This study was part of a larger national survey dataset examining psychological aspects of COVID-19 and its impact on various areas of functioning, with two previously related but distinct (in regard to a priori research questions and hypotheses) published manuscripts examining sex differences in associations between media exposure and COVID-19 anxiety [14], and sex differences in associations between COVID-19 anxiety and cognition [13]. Middle-aged and older adults (*n* = 277; 124 women/153 men; *M*_age_ = 64.68 years, *SD*_age_ = 7.87) living in the United States were recruited via Qualtrics survey panels in July–August 2020. Qualtrics survey panels is an online access panel of individuals willing to participate in online questionnaires. It is considered to be a national online data aggregator. Qualtrics survey panels uses non-probability sampling and sends an email invitation to its users to participate in an online survey, and implements digital fingerprinting technology and IP address verification to ensure data integrity. Participants who met inclusion criteria read and completed documentation of consent prior to completing the online survey. Inclusion criteria were the following: (i) 50+ years of age, (ii) living in the United States, (iii) reported no cognitive impairment or major neurological disorder (mild cognitive impairment, dementia, or other neurological disorders including epilepsy, Parkinson’s Disease, Epilepsy, etc.), and (iv) had normal or corrected-to-normal (via glasses/contacts or hearing aid) vision and hearing. Exclusion criteria were the following: currently receiving pharmacological and/or non-pharmacological treatment for cognition, substance use, fatigue, or mood; and currently receiving non-pharmacological treatment for sleep (sleep medication was not exclusionary but was recorded and examined as a covariate in analyses). Following completion of the online survey, participants were compensated USD 6.50. Study procedures were approved by the University of Missouri Institutional Review Board.

### 2.2. Measures

#### 2.2.1. Demographics

Participants reported their age (in years), sex (0 = male, 1 = female), and highest level of education obtained. Additionally, participants reported the number of medical conditions they were experiencing from a list of common age-related disorders (e.g., heart disease, cancer, diabetes, chronic pain conditions such as fibromyalgia, arthritis, urinary tract problems, etc.) and reported “yes” or “no” if they used sleep or pain medications. Participants were also asked to best describe their current COVID-19 status (diagnosed with COVID-19, still recovering; previously diagnosed with COVID-19 but now fully recovered; never diagnosed with COVID-19).

#### 2.2.2. COVID-19-Related Anxiety

COVID-19-related anxiety was measured via the Coronavirus Anxiety Scale [25]. Participants provided ratings on a scale from 0 (not at all) to 4 (nearly every day) regarding the degree to which they experienced five items specifically related to the pandemic over the past two weeks (e.g., “I felt dizzy, lightheaded, or faint, when I read or listened to news about the coronavirus.”, “I felt nauseous or had stomach problems when I thought about or was exposed to information about the coronavirus.”, etc.). The total score was calculated as the index of interest. Total scores ranged from 0 to 20, and higher scores corresponded to greater COVID-19-related anxiety. Initial development and testing of the Coronavirus Anxiety Scale demonstrated high internal consistency (Cronbach’s alpha, α = 0.93) and validity, and it has very good discriminative ability to determine individuals who do or do not experience dysfunctional anxiety [25]. In the present sample, there was also a high degree of internal consistency of the items included in the Coronavirus Anxiety Scale (Cronbach’s alpha, α = 0.81).

#### 2.2.3. Subjective Sleep

Aspects of sleep health were evaluated via the Pittsburgh Sleep Quality Index (PSQI [26]). Participants answered questions regarding their sleep habits over the past month [how long (in minutes) it takes them to fall asleep each night, how many hours of sleep did you get, etc.] and the frequency (“not during the past month,” “less than once a week,” once or twice a week,” “three or more times a week”) in which they experienced trouble sleeping and/or daytime dysfunction due to various occurrences (“cannot get to sleep within 30 min,” “having to get up to use the bathroom,” “trouble staying awake while driving, “eating meals or engaging in social activity”, etc.). From the responses, seven component scores (subjective sleep quality ratings, sleep onset latency, sleep efficiency, total sleep time, sleep disturbance, daytime dysfunction, and use of sleep medication) are derived, each scored from 0 to 3, and the component scores are added together to produce a global score (PSQI-Total; range 0 to 21), with higher scores representing worse global sleep quality. Other sleep parameters of interest included total sleep time (in hours), sleep efficiency (percentage from 0–100%), and the daytime dysfunction component score. The subjective sleep quality rating (reported as 0—very good, 1—fairly good, 2—fairly bad, and 3—very bad) was also examined as a sleep outcome of interest as this singular qualitative rating can capture different aspects of sleep compared to the PSQI-Total score and other quantitative estimates (total sleep time and efficiency [26]). In the present sample, there was a high degree of internal consistency of the items included in the PSQI (Cronbach’s alpha, α = 0.80).

### 2.3. Statistical Analyses

Multiple linear regressions were conducted using the PROCESS macro (Model 2 [27]) in SPSS (Version 24). Outcome variables included PSQI-Total score, subjective sleep quality rating, total sleep time (in hours), sleep efficiency (%), and daytime dysfunction component score. Independent variables included COVID-19-related anxiety, sex (coded as 0 = men; 1 = women), age, COVID-19-related anxiety × age interaction, and COVID-19-related anxiety × sex interaction. The following covariates were also included in all models: years of education, use of sleep medications, use of pain medications, COVID-19 status, and number of medical conditions. Significant COVID-19-related anxiety by age interactions were clarified by calculating simple slopes of the association between anxiety and sleep metric for sample-estimated age values calculated as follows: middle-aged adults (1 SD below the mean age, ~57 years old), youngest-older adults (mean age, ~65 years old, and oldest-older adults (1 SD above the mean age, ~73 years old). Analyses were evaluated at an alpha of *p* < 0.05. Effect sizes were estimated by examining R^2^_change_ (for interactions) and regression coefficients [28]. In linear regression models, the unstandardized coefficient for each predictor represents the conditional association between the independent and dependent/criterion variables. Specifically, the change in the dependent/criterion variable is associated with a one-unit increase in the independent variable when all other variables in the model equal zero [27].

## 3. Results

### 3.1. Participant Characteristics

The participant demographics and descriptive values for COVID-19-related anxiety and subjective sleep are provided in Table 1. A total of 277 participants (*M*_age_ = 64.68 years, *SD*_age_ = 7.87; 153 men, 124 women) completed all the measures in the survey and were included in the analyses. Independent t-tests (for continuous variables) and chi-squared tests (for categorical variables) revealed no significant differences among men and women regarding any of the measured variables (*p* > 0.05).

### 3.2. Regression Results

All the full models including PSQI-total score (*p* < 0.001, R^2^ = 0.45)], subjective sleep quality ratings (*p* < 0.001, R^2^ = 0.20), total sleep time (*p* = 0.04, R^2^ = 0.07), sleep efficiency (*p* = 0.001, R^2^ = 0.11), and daytime dysfunction (*p* < 0.001, R^2^ = 0.30) were significant and explained 11–45% of the variance. As shown in Table 2, greater COVID-19-related anxiety was independently associated with higher PSQI-Total score, subjective sleep quality ratings, and daytime dysfunction (i.e., worse sleep). Older age was independently associated with lower PSQI-Total score and lower subjective sleep quality ratings.

**Figure 1:**
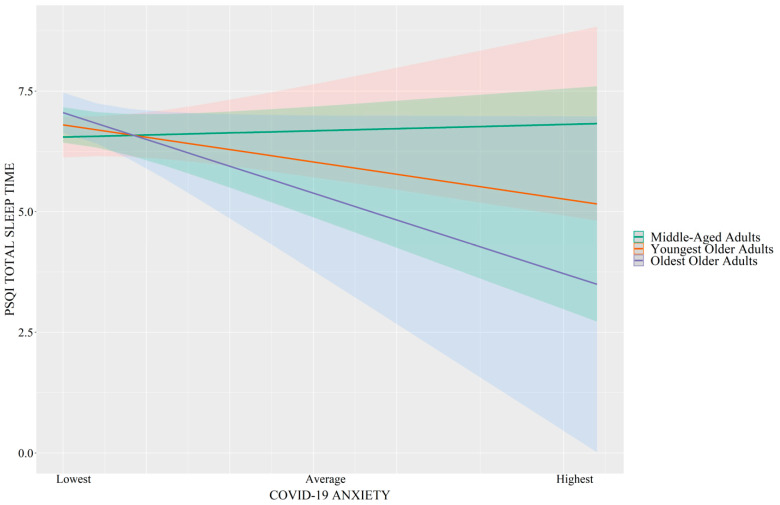
Simple slope regression analyses demonstrating that greater COVID-19-related anxiety is associated with shorter PSQI total sleep time (represented in hours) in oldest-older adults (purple) and youngest-older adults (red) but not middle-aged adults (green). Error bands = 95% CI.

**Figure 2:**
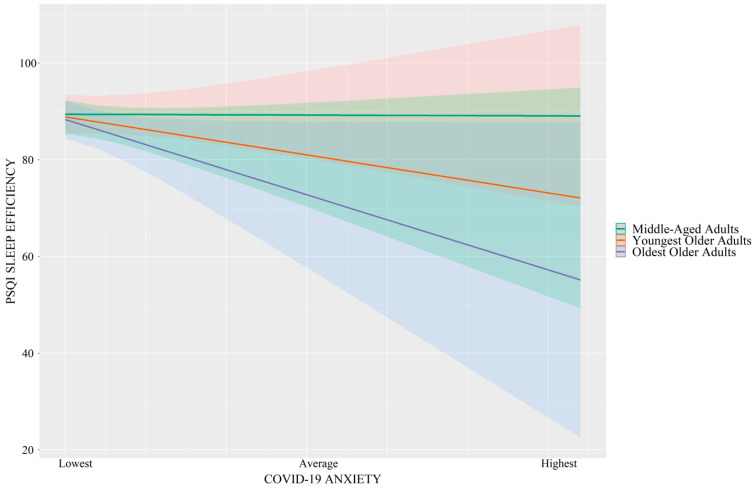
Simple slope regression analyses demonstrating that greater COVID-19 anxiety is associated with lower PSQI-sleep efficiency (represented as a percentage) in oldest-older adults (purple) and youngest-older adults (red) but not middle-aged adults (green). Error bands = 95% CI.

As shown in Table 2, the interaction between COVID-19-related anxiety and age was significantly associated with sleep duration (*p* = 0.01, R^2^_change_ = 0.02) and sleep efficiency (*p* = 0.02, R^2^_change_ = 0.02). These are considered small effect sizes [29,30]. Specifically, as shown in Figure 1, greater COVID-19-related anxiety was associated with shorter total sleep time in oldest-older adults (*B* = −0.25, *SE* = 0.09, *p* = 0.009) and youngest-older adults (*B* = −0.14, *SE* = 0.06, *p* = 0.03) but not in middle-aged adults (*p* = 0.71). Similarly, as shown in Figure 2, greater COVID-19-related anxiety was associated with lower sleep efficiency in oldest-older adults (*B* = −2.71, *SE* = 0.89, *p* = 0.003) and youngest-older adults (*B* = −1.67, *SE* = 0.61, *p* = 0.007) but not in middle-aged adults (*p* = 0.74).

As shown in Table 2, there were no main associations between sex and sleep outcomes. Similarly, interactions between sex and COVID-19-related anxiety were not associated with any sleep outcomes.

## 4. Discussion

The present study evaluated associations between COVID-19-related anxiety and self-reported sleep in middle-aged and older adults living in the United States during the plateau phase of the pandemic (July/August 2020) and examined whether sex or age moderated these associations. The findings showed that greater anxiety related to COVID-19 was associated with several aspects of poor sleep (global sleep quality, subjective sleep quality ratings, and daytime dysfunction). Additionally, age moderated associations between COVID-19 and specific sleep metrics, with only the older adults (~65–85 years of age) showing greater COVID-19-related anxiety associations with shorter sleep duration and lower sleep efficiency. No sex differences were found in the COVID-19-related anxiety and sleep relationship.

Our first hypothesis that greater COVID-19-related anxiety levels would be associated with worse sleep health was generally supported. These findings are consistent with several previous studies across the lifespan finding that worse global sleep quality was linked to worse mental health outcomes related to COVID-19, including anxiety [11], post-traumatic stress [21], and overall negative impact/experience [24]. However, the present results extend the prior findings by showing specific insomnia symptoms that are linked to anxiety tied to COVID-19 in aging adults, including both global and subjective sleep quality ratings, as well as daytime dysfunction. Given the known links between anxiety, hyperarousal, and insomnia [31], it is possible that general 24 h hyperarousal contributed to worse sleep health (reflected by both nighttime and daytime sleep metrics) in the early stages of the pandemic. Further, our age-specific findings suggest that a more dynamic relationship exists between pandemic-related anxiety and sleep health in mid to late life.

Our second hypothesis that higher COVID-19-related anxiety and worse sleep health associations would be strongest in older adults was generally supported, as we observed the expected relationships for total sleep time and sleep efficiency. These findings are inconsistent with some prior work showing no age differences in the mental health and insomnia symptom relationship during early COVID-19 [24]; however, this may be due in part to the older age of the present participant sample (~64 years vs. ~44 years in the prior study), as well as differences in the mental health constructs that were being measured (anxiety in the present study vs. overall perceived negative impact in the prior work [24]). The present findings suggest that older adults may be more vulnerable than middle-aged adults to the anxiety-specific impact of a pandemic on aspects of sleep health related to sleep duration and overall sleep fragmentation. The findings also suggest that oldest-older adults are at the greatest risk as, relative to youngest-older adults, the oldest cohort showed stronger associations between COVID-19-related anxiety and total sleep time (1.8× stronger) and sleep efficiency (1.6× stronger). There are several potential contributing factors to the greater impact of COVID-19-specific anxiety on sleep health in older adults. First, older adults are more vulnerable to COVID-19 morbidity and mortality [32]. Additionally, the level of uncertainty during the early stages of the pandemic (due to the changing health standards and recommendations, shopping and socializing with restrictions, vaccination development, etc. [33]) may have differentially impacted older adults. Furthermore, studies have shown that younger adults are less likely to take COVID-19 precautionary measures compared to their older counterparts [34]. Taken together, it is possible that knowledge regarding age-related vulnerabilities to COVID-19 physiological consequences, uncertainty and changes to daily routines, as well as fear of becoming infected from younger adults could account for the stronger relationship between greater COVID-19-related anxiety and worse sleep health (shorter duration and lower sleep efficiency) in older adults relative to middle-aged adults.

Several age-related physiological mechanisms may also have contributed to the present findings. The increased risk and prevalence of sleep disruption in middle-aged and older adults [35] may exacerbate the existing negative impact of pandemic-related anxiety on total sleep time and sleep efficiency. This was somewhat reflected in our study results as older age was associated with worse global sleep quality and subjective sleep ratings (although age was not associated with total sleep time and sleep efficiency). Thus, anxiety related to COVID-19 may have been particularly problematic for older adults who are already experiencing worse general sleep health. Future work should assess objective measures of sleep to inform how physiological sleep mechanisms (that may correspond to perceptions of sleep quality) are impacted by pandemic-related psychological disruption. As previously stated, greater anxiety has been linked to hyperarousal, both of which are known to be associated with the experience of insomnia symptoms [31]. Given that the autonomic nervous system experiences greater dysfunction with aging (e.g., sustained sympathetic nervous system activation [36]), it is probable that anxiety experienced as a result of COVID-19 is sustained in older adults relative to middle-aged adults and this exacerbates its impact on sleep. The present study examined self-reported anxiety related to COVID-19; therefore, future work is also encouraged to examine age-related physiological responses of the autonomic nervous system (e.g., via non-invasive measures such as cortisol levels and heart rate variability [37]) to inform potential targets for monitoring and/or intervention and optimize future pandemic healthcare for older adults.

Contrary to our third hypothesis, sex did not moderate the COVID-19-related anxiety and sleep health relationship. This is consistent with prior studies examining sex-specific associations of COVID-19 negative impact and generalized anxiety on insomnia severity [24,38]. Unlike prior findings showing women having higher anxiety levels during the early stages of COVID-19 [11,18,19,20], the anxiety levels did not differ between mid-to-late-life men and women in our sample, which could help to explain the lack of a sex-specific impact of worse anxiety on sleep. Further, given that prior studies on sex differences in anxiety during COVID-19 were focused on younger adults [11], our findings may only generalize to aging adults. It is also important to note that prior work [39] in a primarily female sample of older adults has shown associations between anxiety during COVID-19 and other areas of functioning, including participation in recreational activities/hobbies. Given the known differences between older adult men and women across types and patterns of meaningful leisure activities [40], future work may wish to examine whether there are gendered patterns in how these recreational activities impact pandemic-related anxiety and aspects of sleep health.

### 4.1. Practical Implications

When interpreting the present findings and their implications, it is important to consider the context in which the data were collected. Additional research conducted using non-online settings during non-pandemic contexts is needed to determine how well these findings translate to other contexts. Although limited by a relatively small sample, there are several potential clinical and practical implications for current and future pandemic healthcare. First, the main association between COVID-19-related anxiety and sleep suggests that interventions targeted toward alleviating anxiety in the early stages of a pandemic may be particularly beneficial for the general sleep health of all aging adults, and for specific sleep disruptions (shorter total sleep time and lower sleep efficiency) in older adults. For instance, increasing access to behavioral health treatment options (e.g., telehealth) and providing recommendations to maintain mental health during the pandemic [41,42] may help to mitigate poor sleep health and/or insomnia symptoms. Reducing anxiety levels related to this pandemic may also help to decrease overall sleep disturbances and decrease the risk of developing or exacerbating immune system dysfunction and comorbid chronic conditions [43,44], which is critical for older adults who are already at increased risk of these disorders [45]. It may also be important for healthcare providers to reach out to older individuals to provide support and options for anxiety and sleep monitoring and treatment as individuals in this population may be wary of seeking medical assistance [46].

It is important to note that insomnia symptoms can persist in older adults even after the initial precipitating factor (such as anxiety) is no longer present [7,47]. Thus, for older adults, it may be particularly important to not only monitor and/or treat anxiety in the early stages of a pandemic in order to mitigate any negative impacts on sleep health but also continue to monitor sleep and treat disruptions (via first-line treatments such as Cognitive Behavioral Therapy for Insomnia [48]). Effective treatment of sleep disorders is expected to decrease risk of longer-term sleep health-related consequences known to differentially impact older adults, such as increased number of falls [49], Alzheimer’s disease [50], and chronic pain disorders [51].

### 4.2. Limitations

The present study has several limitations. First, the data collection for this study occurred via online Qualtrics panels. Furthermore, the data are self-reported; therefore, there are potential concerns regarding data quality and objectivity. However, recent work has shown that online data collection has psychometric properties similar to traditional/conventional data collection methods [52]. Additionally, several recommended methods for data collection online were followed, including administering pre-screening questions and tracking IP addresses to ensure that there were not multiple responses per participant [52]. Moreover, although sleep data were collected through self-reports and should also be assessed using objective measures (such as actigraphy and polysomnography), we used a standardized questionnaire (PSQI) that is widely used clinically to diagnose insomnia [53]. Additionally, although part of the inclusion criteria was the absence of cognitive impairment, this was verified only via self-report. Further work is encouraged to confirm cognitive status self-report with validated objective global cognitive assessment scales (e.g., Montreal Cognitive Assessment [54]).

Second, we did not collect data regarding participants’ occupation change or job loss during COVID-19, and thus future studies should examine or control for other anxiety-provoking factors (e.g., job loss [55]) that may impact sleep health. Third, although we did not evaluate levels of physical activity in the present study, it is possible that COVID-19-related closures of areas for recreational physical (e.g., parks and gyms) and social (e.g., volunteer centers) activity may have impacted physical and social activity levels and this could in turn have influenced anxiety and sleep levels in aging adults [12]. Thus, future work is encouraged to investigate how changes in physical and/or social activity during a pandemic may alter the anxiety and sleep relationship in older adults.

Fourth, we did not compare patterns of association to younger adults as our focus was on the aging population where there is limited research. However, given research showing associations between generalized anxiety control and sleep in both younger and older individuals [56], the present results should be compared to patterns of COVID-19-related anxiety and sleep in younger adults. Fifth, the study was conducted with participants located only in the United States, which may not generalize to other countries where the impact of COVID-19 (including increased duration of isolation and greater level of restrictions to everyday life) may have differed. Sixth, the findings are based on a relatively small sample and cross-sectional associations. Therefore, future prospective studies in larger samples of aging adults are needed to determine the direction of COVID-19-related anxiety and sleep associations as well as changes in these patterns over time. It should also be noted that, although a formal a priori power analysis was not conducted, the present study did adhere to even the most conservative rule-of-thumb recommendations regarding adequate sample sizes for multiple regression analysis (i.e., 15–25 participants for every predictive model term (independent variable and covariates; 10 in the present study) [57]), thus exceeding the recommendations of minimum sample sizes of 150–250.

Finally, our sample was primarily White/European American. Given the increased risk of morbidity and mortality related to COVID-19 in Black/African American individuals [58], future research should attempt to examine the impact of COVID-19-related anxiety and sleep in a more racially and ethnically diverse sample.

## 5. Conclusions

In middle-aged and older adults living in the United States during the plateau phase of COVID-19 (July/August 2020), greater COVID-19-related anxiety was generally associated with several aspects of worse sleep health (global sleep quality, subjective sleep quality ratings, and daytime dysfunction). Age moderated the anxiety/sleep relationship such that greater COVID-19-related anxiety was only associated with shorter sleep duration and lower sleep efficiency in the oldest age cohorts (~65–85 years of age). No sex differences were found in the COVID-19-related anxiety and sleep relationship. Future pandemic healthcare should prioritize screening and treatment for psychological and sleep-related pandemic effects (in addition to physical health), particularly among older adults.

## Figures and Tables

**Table 1:** Participant characteristics.

	Total (*n* = 277)		Men (*n* = 153)		Women (*n* = 124)	
Variable	Mean (SD)	Range	Mean (SD)	Range	Mean (SD)	Range
Age	64.68 (7.87)	50.00–85.00	64.34 (8.07)	50.00–85.00	65.10 (7.62)	50.00–83.00
Race (*n*, %)						
White/European American	244 (88%)	--	133 (87%)	--	111 (90%)	--
Black/African American	20 (7%)	--	12 (8%)	--	8 (7%)	--
Asian	8 (3%)	--	5 (3%)	--	3 (2%)	--
American Indian/Alaskan Native	1 (1%)	--	1 (1%)	--	0 (0%)	--
Other	4 (1%)	--	2 (1%)	--	2 (2%)	--
Education (*n*, %)						
Some High School	(4.00, 1.40%)	--	(2.00, 1.3%)	--	(2.00, 1.60%)	--
Graduated High School	(53.00, 19.10%)	--	(28.00, 18.30%)	--	(25.00, 20.20%)	--
Some College	(98.00, 35.40%)	--	(55.00, 35.90%)	--	(43.00, 34.70%)	--
Graduate College	(81.00, 29.20%)	--	(41.00, 26.80%)	--	(40.00, 32.30%)	--
Graduate or Professional School	(40.00, 14.40%)	--	(27.00, 17.60%)	--	(13.00, 10.50%)	--
Other	(1.00, 0.40%)	--	(0.00, 0.00%)	--	(1.00%, 0.80%)	--
# of Medical Conditions	2.21 (2.15)	00.00–11.00	2.41 (2.18)	0.00–11.00	1.96 (2.09)	0.00–9.00
Pain Meds Use (*n*, %)						
No	(116.00, 41.90%)	--	(64.00, 41.80%)	--	(52.00, 41.90%)	--
Yes	(161.00, 58.1%)	--	(89.00, 58.20%)	--	(72.00, 58.10%)	--
Sleep Meds Use (*n*, %)				--		--
No	(212.00, 76.50%	--	(114.00, 74.50%)	--	(98.00, 79.00%)	--
Yes	(65.00, 23.50%	--	(39.00, 25.50%)	--	(26.00, 21.00%)	--
COVID-19 Exposure (*n*, %) ^a^						
Diagnosed, Still Recovering	(1.00, 0.40%)	--	(0.00, 0.00%)	--	(1.00, 0.80%)	--
Previously Diagnosed, Fully Recovered	(1, 0.40%)	--	(1.00, 0.70%)	--	(0.00, 0.00%)	--
Never Diagnosed	(275.00, 0.40%)	--	(152.00, 99.3%)	--	(123.00, 99.20%)	--
COVID-19-Related Anxiety	0.84 (2.10)	0.00–16.00	0.80 (2.04)	0.00–16.00	0.89 (2.18)	0.00–15.00
Subjective Sleep (PSQI)						
Total Score	6.26 (3.99)	0.00–18.00	6.59 (4.18)	0.00–18.00	5.85 (3.71)	0.00–16.00
Subjective Sleep Quality Ratings ^b^	1.13 (0.78)	0.00–3.00	1.19 (0.80)	0.00–3.00	1.07 (0.75)	0.00–3.00
Total Sleep Time (hours)	6.73 (1.60)	0.00–15.00	6.65 (1.72)	2.00–15.00	6.83 (1.44)	0.00–10.00
Sleep Efficiency (%) ^c^	85.82 (15.28)	0.00–100.00	85.45 (15.28)	0.00–100.00	86.28 (15.33)	0.00–100.00
Daytime Dysfunction	0.65 (0.74)	0.00–3.00	0.69 (0.76)	0.00–3.00	0.59 (0.70)	0.00–3.00

Note: # = Number; Meds = medications; ^a^ *n* = 275, *n*_Men_ = 152, *n*_Women_ = 123; ^b^ *n* = 274, *n*_Men_ = 152, *n*_Women_ = 122; ^c^ *n* = 270, *n*_Men_ = 148, *n*_Women_ = 122.

**Table 2:** Multiple regression results of associations between COVID-19-related anxiety and self-reported sleep (via the PSQI) in middle-aged and older adults living in the United States in July/August 2020.

	PSQI-Total Score				Subjective Sleep Quality Ratings				Total Sleep Time				Sleep Efficiency				Daytime Dysfunction			
	*B*	*SE*	*t*	*p*	*B*	*SE*	*t*	*p*	*B*	*SE*	*t*	*p*	*B*	*SE*	*t*	*p*	*B*	*SE*	*t*	*p*
COVID-19 Anxiety	0.68	0.15	4.64	<0.001	0.11	0.03	3.18	0.002	−0.10	0.08	−1.29	0.20	−1.05	0.74	−1.41	0.16	0.19	0.03	6.19	<0.001
Age	−0.06	0.02	−2.58	0.01	−0.02	0.01	−3.79	<0.001	0.02	0.01	1.61	0.11	−0.17	0.12	−1.48	0.14	−0.01	0.005	−1.94	0.05
COVID-19 Anxiety x Age	0.02	0.01	1.55	0.12	0.003	0.003	1.01	0.31	−0.02	0.006	−2.47	0.01	−0.13	0.06	−2.26	0.02	0.002	0.002	0.75	0.45
Sex	−0.52	0.37	−1.43	0.15	−0.09	0.09	−1.02	0.31	0.15	0.20	0.74	0.46	0.68	1.85	0.37	0.71	−0.08	0.08	−1.04	0.30
COVID-19 Anxiety x Sex	−0.09	0.21	−0.44	0.66	−0.05	0.05	−1.14	0.25	−0.03	0.12	−0.29	0.77	−0.99	1.11	−0.90	0.37	−0.07	0.04	−1.56	0.12
Education	0.21	0.18	1.16	0.25	−0.03	0.04	−0.60	0.55	−0.09	0.10	−0.93	0.35	−0.75	0.94	−0.79	0.43	0.03	0.04	0.70	0.48
# of Medical Conditions	0.29	0.10	2.91	0.004	0.04	0.02	1.59	0.11	−0.04	0.05	−0.77	0.44	−0.31	0.50	−0.63	0.53	0.04	0.02	1.99	0.05
Sleep Meds Use	3.70	0.45	8.16	<0.001	0.29	0.11	2.69	0.01	−0.38	0.25	−1.55	0.12	−4.74	2.31	−2.06	0.04	0.20	0.10	2.08	0.04
Pain Meds Use	0.65	0.41	1.61	0.11	0.14	0.10	1.40	0.16	0.04	0.22	0.18	0.85	−2.71	2.04	−1.33	0.18	0.08	0.09	0.90	0.37
COVID-19 Status	2.85	1.53	1.86	0.06	0.57	0.36	1.57	0.12	−2.05	0.97	−2.11	0.04	−19.03	9.11	−2.09	0.04	0.33	0.32	1.02	0.31

Note: # = Number; PSQI = Pittsburgh Sleep Quality Index. All sleep outcomes are based on self-reported values from the PSQI. Meds = medication.

## Data Availability

Data can be requested by contacting the PI and corresponding author of the study (PI Curtis). The data are not publicly available due to additional planned research projects that are utilizing this dataset. This study was not preregistered.
